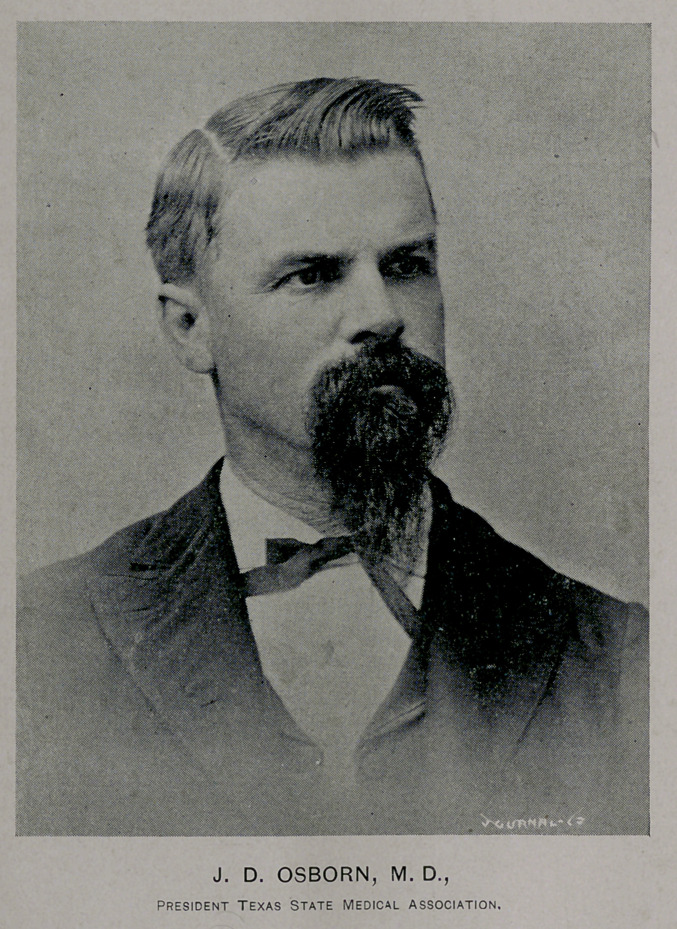# Biographical Sketch of Dr. Osborn

**Published:** 1892-05

**Authors:** 


### JAMES D. OSBORN, M. D., CLEBURNE, TEXAS.

### PRESIDENT-ELECT TEXAS STATE MEDICAL ASSOCIATION.

Dr. Osborn was born August 24th, 1845, in Green county, Alabama. Graduated from Southern University, Greensboro, Alabama, in July, 1862, and left the rostrum for the "tented field." Served with the gallant Forrest until the fall of 1864, where he was severely wounded at Columbia, Tennessee, while adjutant of the 7th Alabama Cavalry, Rucker's Brigade.

After the surrender, he studied medicine under his father, Dr. T. C. Osborn, in Greensboro, Ala., until September, 1865. He then entered the University of Virginia and graduated July 2nd, 1866; practiced medicine in Greensborough until 1874; attended lectures in New Orleans at Medical Department of Louisiana University, and Hospital Clinics at Charity Hospital, under Stone, Bemis, Richardson, Chaille, etc., during winters of '67, '68 and '69; was elected Professor of Surgery in Southern University in 1870.

He adopted Texas as his home in 1875, and has been practicing in Cleburne continuously for 17 years—since Feb., 1875.

He was married in 1870, in Greensboro, Ala., to Miss Julia K. Pittman. Of this union there are four children—two boys and two girls—oldest 21 years and youngest 10 years of age. The youngest are Texans, and glory in their State.

## Figures and Tables

**Figure f1:**